# Oxymatrine exerts protective effects on osteoarthritis via modulating chondrocyte homoeostasis and suppressing osteoclastogenesis

**DOI:** 10.1111/jcmm.13674

**Published:** 2018-05-25

**Authors:** Yafei Jiang, Weilin Sang, Cong Wang, Haiming Lu, Tao Zhang, Zhuoying Wang, Yu Liu, Bao Xue, Song Xue, Zhengdong Cai, Yingqi Hua, Libo Zhu, Jinzhong Ma

**Affiliations:** ^1^ Department of Orthopedics Shanghai General Hospital Shanghai Jiao Tong University School of Medicine Shanghai China; ^2^ Shanghai Bone Tumor Institution Shanghai China

**Keywords:** cartilage, inflammation, osteoarthritis, oxymatrine, subchondral bone

## Abstract

Osteoarthritis (OA) is a common degenerative disease characterized by the progressive destruction both articular cartilage and the subchondral bone. The agents that can effectively suppress chondrocyte degradation and subchondral bone loss are crucial for the prevention and treatment of OA. Oxymatrine (OMT) is a natural compound with anti‐inflammatory and antitumour properties. We found that OMT exhibited a strong inhibitory effect on LPS‐induced chondrocyte inflammation and catabolism. To further support our results, fresh human cartilage explants were treated with LPS to establish an ex vivo degradation model, and the results revealed that OMT inhibited the catabolic events of LPS‐stimulated human cartilage and substantially attenuated the degradation of articular cartilage ex vivo. As subchondral bone remodelling is involved in OA progression, and osteoclasts are a unique cell type in bone resorption, we investigated the effects of OMT on osteoclastogenesis, and the results demonstrated that OMT suppresses RANKL‐induced osteoclastogenesis by suppressing the RANKL‐induced NFATc1 and c‐fos signalling pathway in vitro. Further, we found that the anti‐inflammatory and anti‐osteoclastic effects of oxymatrine are mediated via the inhibition of the NF‐κB and MAPK pathways. In animal studies, OMT suppressed the ACLT‐induced cartilage degradation, and TUNEL assays further confirmed the protective effect of OMT on chondrocyte apoptosis. MicroCT analysis revealed that OMT had an attenuating effect on ACLT‐induced subchondral bone loss in vivo. Taken together, these results show that OMT interferes with the vicious cycle associated with OA and may be a potential therapeutic agent for abnormal subchondral bone loss and cartilage degradation in osteoarthritis.

## 1 INTRODUCTION

Osteoarthritis (OA) is a common, heterogeneous arthritic disorder that causes a major public health burden all over the world.^1^ According to previous studies, OA ranks 11th of the 291 diseases listed by the WHO and is one of the leading causes of global disability.^2^ Despite the high prevalence of OA, the specific pathogenesis of the disease is not yet clear.

Over the last decade, our understanding of OA has improved dramatically. The disease is now considered an inflammatory disease,^3^ characterized by a low‐grade inflammatory state.^4^ Pro‐inflammatory cytokines not only stimulate their own expression but also activate the NF‐κB and mitogen‐activated protein kinase (MAPK) signalling pathway, the key mediators whose activation leads to the induction of cartilage matrix‐degrading enzymes such as a disintegrin and metalloproteinase with thrombospondin motifs (ADAMTS), matrix metalloproteinases (MMPs) and aggrecanases.^5^, ^6^, ^7^ Furthermore, OA is now considered an organ‐level disease that involves not only the articular cartilage but also the subchondral bone,^8^, ^9^ characterized by a decrease in bone density that is later followed by abnormal bone deposition. Several studies have suggested that there is increased porosity in the subchondral plate during disease development, as evidenced by a mouse model of instability‐induced knee OA.^10^, ^11^ Osteoclasts, as the main cells for bone resorption, have recently become a new target for the treatment of osteolytic diseases.^12^, ^13^, ^14^, ^15^, ^16^ For this reason, treatment focusing on the prevention of cartilage degeneration and osteoclast‐mediated bone loss is of great significance for the prevention and treatment of OA.

In recent years, there has been increasing interest in compounds extracted from traditional Chinese medicinal plants, which may play a significant role in the treatment of OA because of their potent anti‐inflammatory effects.^17^ Oxymatrine (OMT), which is a quinolizidine alkaloid extracted from the traditional Chinese herb *Sophora flavescens* Aiton,^18^ has attracted much attention because of its low toxicity and wide pharmacological effects and is now often used to treat various diseases, including angina pectoris, myocardial infarction, stroke, cancer and inflammation.^19^, ^20^, ^21^ The anticancer effects of OMT mainly through inhibiting cancer proliferation, invasion and metastasis.^18^, ^21^ Furthermore, OMT accelerates cancer cell apoptosis and reverses multidrug resistance when combined with other chemotherapeutic drugs.^22^ The anti‐inflammation effects of OMT appear to involve both inhibition of MAPK phosphorylation and reduce the activation of the NF‐κB signalling pathway.^23^, ^24^, ^25^, ^26^ Furthermore, OMT exhibits substantial therapeutic potential for the treatment of myocardial diseases through modulating of the JAK2/STAT3 and TGF‐β1/Smad3 signalling pathway,^27^, ^28^ but the effects of OMT on the onset and progression of OA have not been reported, to our knowledge.

In this study, we investigated the potential effect of OMT as a preventive treatment for OA in vitro and in vivo. We are committed to providing OMT as a novel potential alternative for the treatment of OA.

## 2 MATERIALS AND METHODS

### 2.1 Reagents and cell lines

Purified oxymatrine (MF: C_15_H_24_N_2_O_2_, MW: 264.367, purity > 98%) was purchased from Shanghai Yuan Ye Biotechnology Co. Ltd. (Shanghai, China). Penicillin, streptomycin, trypsin‐EDTA, RIPA buffer, protease inhibitor cocktail, phosphatase inhibitor cocktail PBS, TBS tartrate‐resistant acid phosphate (TRAP) and foetal bovine serum (FBS) were purchased from Sigma‐Aldrich Chemicals Private Limited (St. Louis, MO, USA). Antibodies against p‐p65, p65, ERK, JNK, P38, P‐ERK, P‐JNK, P‐P38 and IκBɑ were obtained from Cell Signaling Technology (Danvers, MA, USA). Histones H3 and GAPDH antibodies were purchased from Abcam (Hong Kong, China). β‐actin antibodies were purchased from Santa Cruz Biotechnology (Santa Cruz, CA). All cell lines were obtained from the American Type Culture Collection (ATCC, Manassas, VA) and grown in the media recommended by ATCC.

### 2.2 Primary human chondrocyte isolation and culture

Osteoarthritis cartilage tissues were obtained from patients who underwent total knee arthroplasty. Cartilage was separated from the underlying bone and connective tissues, cut into 1 × 1 × 1 mm^3^ pieces and washed 3 times with PBS. Afterwards, the joint cartilage pieces were digested with 0.25% trypsin‐EDTA solution for 30 minutes at 37°C, followed by collagenase type II for 6 hours at 37°C. Then, the samples were centrifuged at 300 g for 5 minutes, and the supernatant was discarded. The chondrocytes were collected and cultured in DMEM/F12 medium containing 10% FBS and 100 U/mL of penicillin‐streptomycin at 37°C in a humidified atmosphere of 5% CO_2_. Only passages 2‐4 were used in our study to avoid the phenotype loss. All patients underwent surgery at the Department of Orthopaedics of the Shanghai General Hospital of Shanghai JiaoTong University School of Medicine (Shanghai, China) between 2016 and 2017. The present research was approved by the Institutional Research Ethics Committee of Shanghai General Hospital, and informed consent was obtained from all patients.

### 2.3 Cell viability

The effect of OMT on cell viability was assessed using a cell counting kit 8 (CCK‐8) assay (Dojindo, Kumamoto, Japan) according to the manufacturer's instructions. Human OA chondrocytes and BMMs were cultured in 96‐well plates at a density of 5 × 10^3^ cells per well and then pre‐treated with or without different concentrations (0, 0.5, 1, 2 or 4 mg/mL) of OMT for the indicated time. After that, 10 μL of CCK‐8 was added to each well and incubated at 37°C for 2 hours. The optical density was read at a wavelength of 450 nm with a microplate spectrophotometer (SpectraMax; Molecular Devices, Sunnyvale, CA). Three independent experiments were carried out in triplicate.

### 2.4 Real‐time PCR analysis

After treatment at the indicated time‐points, total RNA was extracted from chondrocytes using TRIzol reagent (Invitrogen, CA, USA) according to the manufacturer's instructions. For each sample, reverse transcription was accomplished on 1 ng of total RNA using the M‐MLV reverse transcriptase (Invitrogen). PCR assays were performed in triplicate on a ViiA™7 real‐time PCR system (Life Technology, USA) according to the manufacturer's instructions. The mRNA expression levels of target genes were normalized to that of β‐actin, and the 2^−ΔΔCt^ method was used to assess the relative expression of different candidate genes. The primers used for real‐time PCR are listed in Table S1.

### 2.5 Human cartilage explant ex vivo assay

Fresh articular cartilage was collected aseptically from patients who underwent total knee arthroplasty for primary knee OA. Explants were dissected from the articular surface without calcified cartilage layers; 2‐mm‐thick explants with a 4‐mm diameter were obtained, washed in PBS 3 times and cultured in DMEM/high glucose supplemented with 10% FBS. The explants were exposed with 10 μg/mL lipopolysaccharides (LPS) in the presence or absence of OMT to induce inflammatory and catabolic damage in the cartilage. The medium, with fresh LPS and OMT, was changed every 3 days. After 7 and 14 days of treatment, the explants were fixed in 4% PFA for 24 hours, embedded in paraffin, sectioned into 4‐μm‐thick sections and stained with Safranin O for histological analysis and with collagen II for immunohistochemical analysis. The culture media from days 3‐7 and days 11‐14 were collected to analyse glycosaminoglycan (GAG) release with the dimethylmethylene blue (DMMB) assay according to the manufacturer's instructions.

### 2.6 ELISA

MMPs and pro‐inflammatory cytokines, like MMP2, MMP9, MMP13, IL‐6 and TNF‐α, are believed to mediate the progression of OA. The chondrocyte supernatants from different groups were collected and were analysed using ELISA kits (R&D Systems, Minneapolis, MN, USA) according to the manufacturer's instructions. The results are expressed as picograms per millilitre.

### 2.7 In vitro osteoclast differentiation assay

For the in vitro osteoclastogenesis assay, bone marrow cells (BMMs) were isolated from the femurs and tibias of C57BL/6 mice and cultured in α‐MEM supplemented with 10% FBS and macrophage colony‐stimulating factor (M‐CSF) (10 ng/mL) for 24 hours; the BMMs were allowed to adhere overnight and then cultured with mM‐CSF (30 ng/mL) and Receptor Activator for Nuclear Factor‐κ B Ligand (RANKL) (50 ng/mL) in 96‐well plates in the presence or absence of OMT for 6 days with media changed every 2 days. After treatment, cells were fixed with 4% paraformaldehyde (PFA) for 20 minutes at room temperature and stained for TRAP activity according to the manufacturer's instructions. TRAP‐positive multinucleated cells with more than 3 nuclei were counted as osteoclasts.

### 2.8 In vitro osteoblast differentiation assay

BMSCs between passages 3 and 5 were used for experiments. To induce osteogenic differentiation, BMSCs were cultured with a‐MEM supplemented with 10% FBS, ascorbic acid (50 mg/mL) and β‐glycerophosphate (10 mmol/L) for up to 7 and 14 days, after treatment, the cells were fixed with 4% PFA for 20 minutes at room temperature and stained for Alizarin Red according to the manufacturer's instructions.

### 2.9 Western blot

After treatment, total protein was extracted from chondrocytes using ice‐cold RIPA lysis buffer. The protein concentration was determined using a BCA protein assay kit (Beyotime, NanJing, China). Total protein (20‐50 μg) was resolved on 10% SDS‐PAGE and transferred to PVDF membranes. Membranes were blocked with 5% non‐fat milk in TBS containing 0.1% Tween‐20 (TBS‐T) for 1 hour and then incubated with the primary antibodies overnight at 4°C. After washing 3 times with TBS‐T for 10 minutes, the membranes were incubated with HRP‐conjugated secondary antibodies (1:2000) for 1 hour at room temperature. Finally, membranes were developed by an enhanced chemiluminescence (ECL) kit and a Bio‐Rad ChemiDoc MP Imaging System.

### 2.10 Immunofluorescence microscopy

After treatment, cells were fixed with 4% paraformaldehyde (PFA) for 20‐30 minutes at room temperature. The cells were permeabilized with 0.1% Triton X‐100 for 15 minutes. The cells were blocked in 0.1% bovine serum albumin (BSA) for 30 minutes and then incubated with primary antibody at 4°C overnight. After washing 3 times for 5 minutes with a TBS‐T solution, Alexa Fluor‐conjugated secondary antibodies (1:400) were used for visualization. Cell nuclei were stained with DAPI for 3 minutes. After washing 3 times for 5 minutes, the dish was covered with PBS. The images were collected on an Olympus FluoView FV10i confocal microscope.

### 2.11 Nuclear and cytoplasmic extraction

Human primary chondrocytes and BMMs were treated with OMT for 2 hours and then stimulated with LPS or RANKL for another 30 minutes. To separate the cytoplasmic and nuclear proteins, cell pellets were processed using a nuclear and cytoplasmic extraction kit (Beyotime, NanJing, China) according to the manufacturer's instructions.

### 2.12 Mice OA models and treatment

Surgically induced joint destabilization by anterior cruciate ligament transection (ACLT) was used to study the pathogenesis of cartilage destruction and to evaluate the therapeutic potential for treating OA with OMT. Ten‐week‐old C57BL/6 male mice were purchased from the Animal Center of the Chinese Academy of Sciences, Shanghai, China, and were housed in specific pathogen‐free facilities at the laboratory animal unit of the Shanghai General Hospital. The experimental mice were subjected to surgically induced OA by anterior cruciate ligament transection (ACLT) using a needle in the right knee without opening the joint, and the anterior drawer test was carried out to ensure that the anterior cruciate ligament had been completely transected. A sham operation was performed in the right knee joint of mice in the control group. Mice were maintained on a 12‐hours light/dark cycle under a constant temperature of 24 ± 2°C and a relative humidity of 55% ± 5%; they were allowed free access to food and water. The mice were allowed to move freely in the cages. After surgery, the mice were randomly divided into 4 groups. Group I (sham group, n = 5): mice did not undergo surgery or receive treatment. Group II (ACLT + vehicle, n = 5): mice underwent ACLT and received intraperitoneal injections of PBS (25 mg/kg, daily). Group III (ACLT + low‐dose OMT, n = 5): mice underwent ACLT and received intraperitoneal injections of low‐dose OMT (25 mg/kg, daily). In Group IV (ACLT + high‐dose OMT, n = 5), the mice underwent ACLT surgery and received intraperitoneal injections of OMT (50 mg/kg, daily). All procedures were reviewed for consideration of animal welfare and were approved by the ethics committee of the Shanghai General Hospital.

### 2.13 Histological assessments

All animals were killed at 6 weeks after surgery. Knee joint samples were collected, fixed in 4% PFA for 24 hours, decalcified in 10% EDTA at 4°C for several days, embedded in paraffin and sectioned into 4‐μm‐thick sections. The sections were stained with haematoxylin & eosin (H&E) and Safranin O/Fast green staining for further histological analyses. Histopathological features were semiquantitatively scored according to the Osteoarthritis Research Society International (OARSI) grading system.

### 2.14 TUNEL staining

To detect apoptotic cells in articular cartilage, sections were stained by terminal deoxynucleotidyl transferase dUTP nick end labelling (TUNEL) according to the manufacturer's instructions. The cell nuclei were stained with DAPI. The percentage of apoptosis in cartilage was calculated by the ratio of the number of TUNEL‐positive cells to DAPI‐stained cells.

### 2.15 MicroCT analysis

After treatment, all hind limbs of the mice were dissected and fixed in 4% PFA for 24 hours before micro CT analysis. Axial scans were performed using a high‐resolution μCT (70 kv, 200 μA) from the YUEBO Company (Hangzhou, China). Then, sagittal slices were reconstructed at a resolution of 10 μm. Subchondral bone under the tibial plateau region was defined as region of interest (ROI). Trabecular bone was analysed including bone mineral density (BMD), bone volume/total tissue volume (BV/TV), trabecular number (Tb.N), trabecular thickness (Tb.Th), trabecular separation (Tb.Sp) and Structure Model Index (SMI) using the manufacturer provided software.

### 2.16 Statistical analysis

Statistical analysis was performed with one‐way ANOVA. Data are shown as the means ± SD. Statistical calculations were performed by SPSS 17.0. *P* < .05 was considered to indicate a significant difference from the control.

## 3 RESULTS

### 3.1 OMT inhibited LPS‐induced pro‐inflammatory cytokines and matrix metalloproteinase (MMP) production in human OA chondrocytes

**Figure 1:**
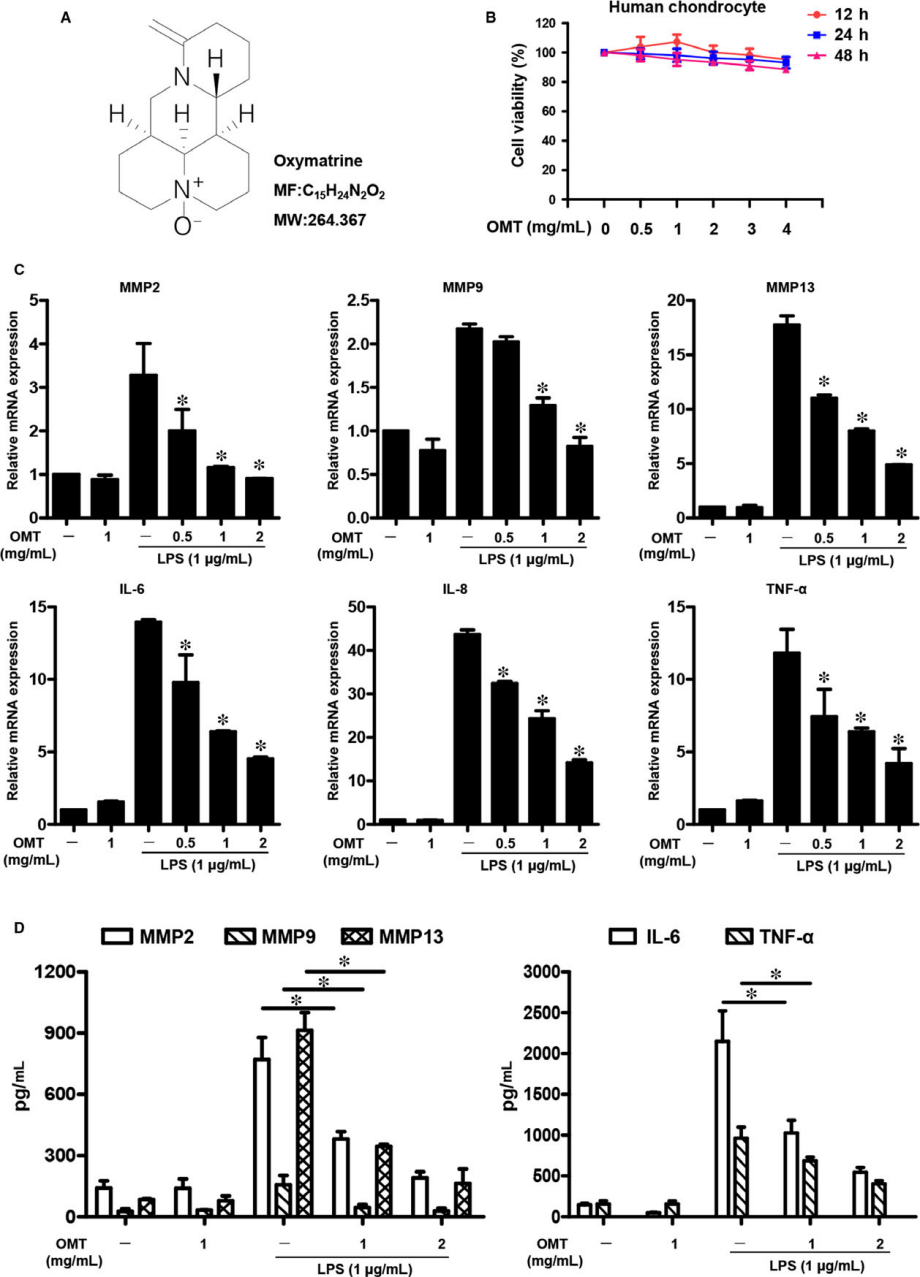
Treatment with OMT protects chondrocytes from LPS‐induced inflammation and catabolism. A, Chemical structure of OMT. B, The viability of chondrocytes was evaluated using the CCK‐8 assay. C, Pro‐inflammatory cytokines, including IL‐6, ‐8, ‐10 and TNF‐α, were assessed by real‐time PCR. D, The expression levels of MMP2, MMP9, MMP13, IL‐6 and TNF‐α were investigated using ELISA. Data are shown as the means ± SD of triplicate independent experiments. *P <* *.05 compared to the control group

To explore the effects of OMT (Figure 1A) on chondrocytes, we first investigated the effect of OMT exposure on the viability of primary human chondrocytes. As shown in Figure 1B, cell viability decreased with OMT exposure up to 2 mg/mL, but this compound had no significant toxicity on chondrocyte viability.

Lipopolysaccharides has been used as a classical methodological approach to perform diverse in vitro studies and has been used to induce arthritis.^29^ In our studies, we have investigated the effects of OMT on the production of pro‐inflammatory cytokines and MMPs in LPS‐stimulated human OA chondrocytes. As shown in Figure 1C, the real‐time PCR results showed that LPS exposure significantly increased the production of cytokines (IL‐6, ‐8 and TNF‐α) and MMPs (MMP‐2, ‐9 and ‐13) compared with the unstimulated controls. However, pre‐treatment with OMT markedly prevented LPS‐stimulated pro‐inflammatory cytokine and MMP production. To further validate the above results, we also assessed the effects of OMT on the LPS‐induced protein expression of those MMPs and cytokines using ELISA, and the results further supported the real‐time PCR results (Figure 1D). Collectively, these results indicated that OMT exerts a protective effect on OA by regulating inflammatory reactions and cell catabolism.

### 3.2 OMT attenuates the LPS‐induced degeneration of human articular cartilage explants

**Figure 2:**
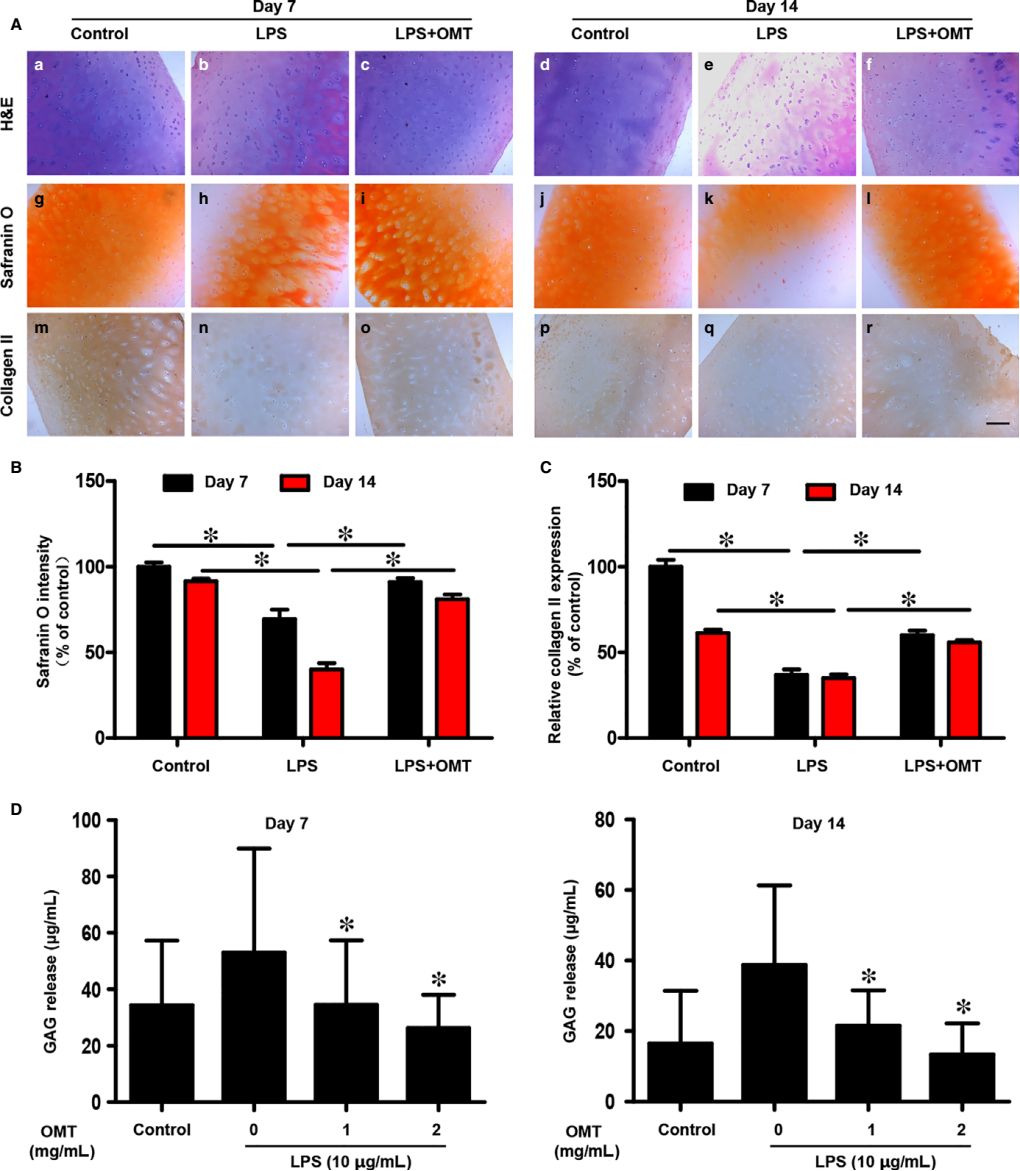
Effects of OMT on human articular cartilage degradation in an ex vivo model. A, Human cartilage explants were stained with H&E (a‐f) and Safranin O (g‐l) Immunohistochemical staining for collagen II (m‐r). Scale bar, 100 μm. B, The overall Safranin O staining intensity of the explants was assessed. C, The panels show the quantification of collagen II immunohistochemical staining. D, The amount of GAGs released into the supernatant was quantified using the DMMB assay. Data are represented as the means ± SD of triplicate independent experiments. *P value < .05 was considered significantly different from the control

To investigate the protective effects of OMT on human articular cartilage, we then examined the LPS‐induced degradation of human articular cartilage ex vivo. As shown in Figure 2A, Safranin O staining showed that OMT administration significantly attenuated the LPS‐induced loss of proteoglycan. The results clearly showed that OMT was able to preserve the proteoglycan content in the ECM of ex vivo explants, as indicated by the red staining intensity being similar to that of the control. Immunohistochemical staining results further revealed that LPS exposure inhibited the expression of collagen II, while in the OMT‐treated group, the expression of collagen II was higher than that of the LPS exposure group. The differences in the overall staining intensity of the explant groups were assessed according to the Safranin O staining results (Figure 2B). Based on the immunostaining intensities of 5 randomly selected areas of cartilage, the expression level of collagen II was calculated and analysed (Figure 2C).

As the changes in LPS‐induced chondrocytes lead to the up‐regulation of MMPs, the GAG content released into the supernatant was interpreted as the degree of matrix degradation caused by MMPs. We measured the concentration of GAGs in the culture medium by the DMMB assay, and the results showed that at days 7 and 14, OMT treatment significantly decreased the release of GAGs from LPS‐stimulated human articular cartilage tissues into the culture supernatant (Figure 2D). Above all, these results demonstrated that OMT inhibited catabolic events in LPS‐stimulated human cartilage and substantially attenuated the degradation of articular cartilage.

### 3.3 OMT inhibited RANKL‐induced osteoclast formation in vitro

**Figure 3:**
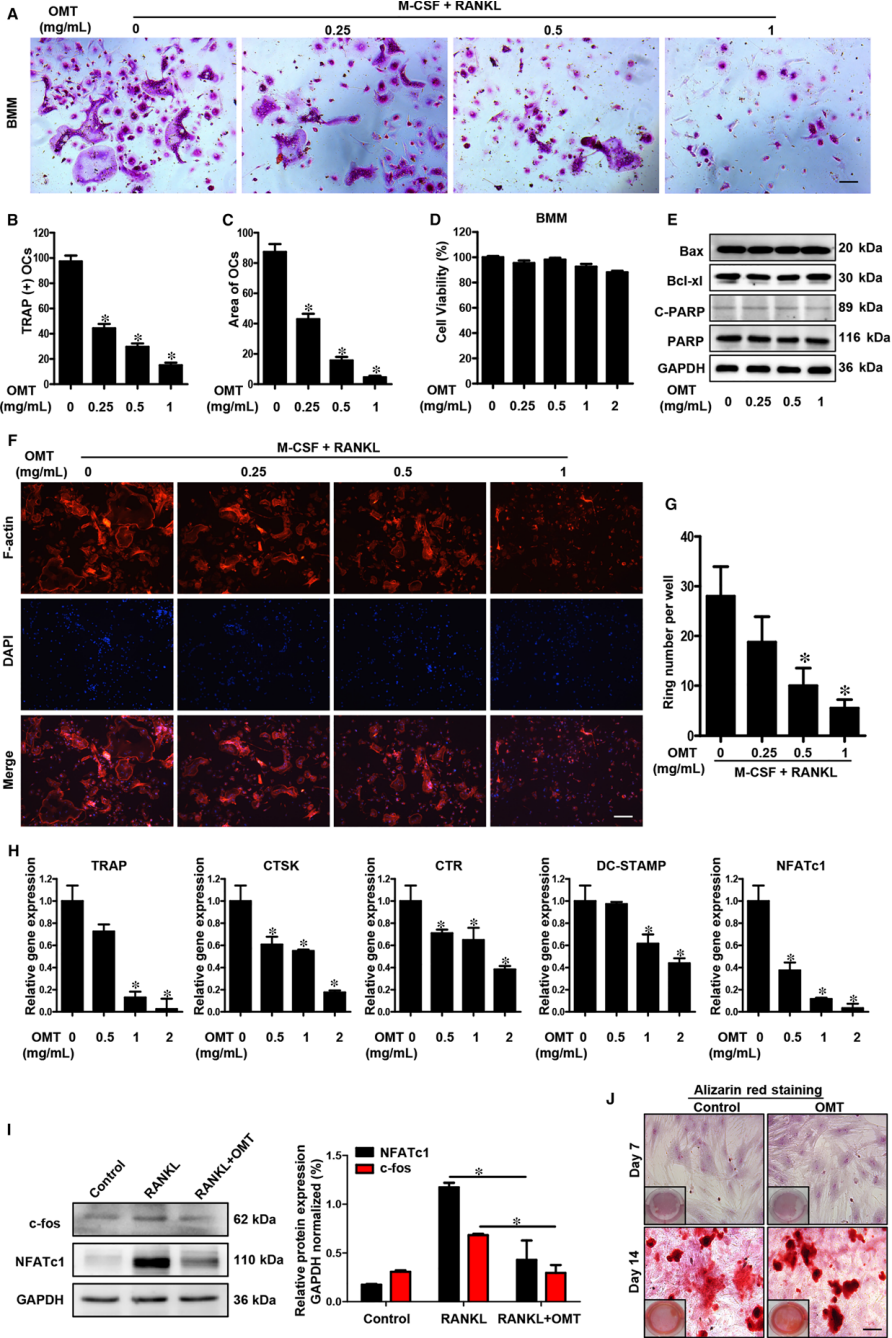
OMT dose‐dependently inhibited osteoclastogenesis in vitro. A, The effect of OMT on mouse BMM differentiation was detected using TRAP staining. Scale bar, 100 μm. The number (B) and area (C) of TRAP‐positive multinucleated (≥3 nuclei) osteoclasts were quantified. D, The effect of OMT on cell viability of mouse BMMs was measured by CCK‐8 assay. E, The effects of OMT on apoptotic‐related proteins were analysed using Western blotting; GAPDH was used as the loading control. F, After differentiation, BMMs were fixed and stained for F‐actin ring. Scale bar, 100 μm. G, The number of osteoclasts per well with an intact actin ring was counted. H, The expression of OC marker genes (CTSK, NFATc1, TRAP, DC‐STAMP and CTR) was determined using real‐time PCR. The gene expression was normalized to that of β‐actin. I, OMT suppresses the RANKL‐induced activation of NFATc1 and c‐Fos. GAPDH was used as the loading control. (right panel) The protein expression levels of NFATc1 and c‐Fos were quantified using ImageJ. J, The effects of OMT on osteogenesis were detected using Alizarin Red staining. Scale bar, 100 μm. All experiments were performed at least 3 times. Values are expressed as the means ± SD. *P < .05 compared to the control

To investigate the effect of OMT on osteoclastogenesis, we employed an in vitro osteoclast differentiation model; mouse BMMs were stimulated with M‐CSF or RANKL. As shown in Figure 3A, OMT reduced TRAP‐positive multinucleated osteoclasts in a dose‐dependent manner. The number and average area of TRAP‐positive OCs were reduced (Figure 3B,C). To rule out the possibility that OMT induces cytotoxic effects on BMM‐derived OCs, we examined the potential cytotoxicity of OMT on BMMs with the CCK‐8 assay. Our results indicated that up to 2 mg/mL, this compound had no significant cytotoxic effects on BMMs (Figure 3D). Furthermore, there was no change in the expression levels of the apoptosis‐related proteins Bax or Bcl‐xL or in the activation of the PARP apoptotic pathways (Figure 3E). Our results showed that the effects of OMT on osteoclastogenesis are not mediated by the toxicity of this compound on cell viability.

An essential prerequisite for OC bone resorption is the formation of a dense F‐actin‐rich podosome. Our confocal microscopy results demonstrated that OMT dose‐dependently disrupted both the morphology and number of OC F‐actin rings. As shown in Figure 3F, in the OMT‐treated group, there were significant reductions in the number of F‐actin rings (Figure 3G). Collectively, these findings suggested that OMT impaired bone resorption, at least in part, by disrupting the integrity of both F‐actin ring formation and the cytoskeleton more generally.

To clarify the mechanism of OMT on osteoclastogenesis, real‐time PCR was used to determine the expression of OC marker genes after exposure to RANKL in the presence or absence of OMT. The results showed that OMT dose‐dependently suppressed the expression of OC marker genes including TRAP, cathepsin K (CTSK), NFATc1, dendrite cell‐specific transmembrane protein (DC‐STAMP) and calcitonin receptor (CTR) (Figure 3H). To further examine the effects of OMT on RANKL signalling, we studied its effect on the RANKL‐induced activation of c‐fos and NFATc1 using Western blotting. BMMs were treated with RANKL (50 ng/mL) in the presence or absence of OMT (1 μg/mL) for 3 days, as shown in Figure 3I, RANKL increased the protein expression levels of NFATc1 and c‐fos, while OMT suppressed this effect. Most importantly, OMT inhibited osteoclast formation by suppressing the RANKL‐induced NFATc1 and c‐fos signalling pathway.

To evaluate the influence of OMT on osteoblast differentiation from progenitors, induction cultures of BMSCs were performed. Determination of differentiation was based on the Alizarin Red staining. As shown in Figure 3J, OMT had no significant effects on osteogenic differentiation of BMSC.

### 3.4 OMT suppresses the NF‐κB and MAPK signalling pathway

**Figure 4:**
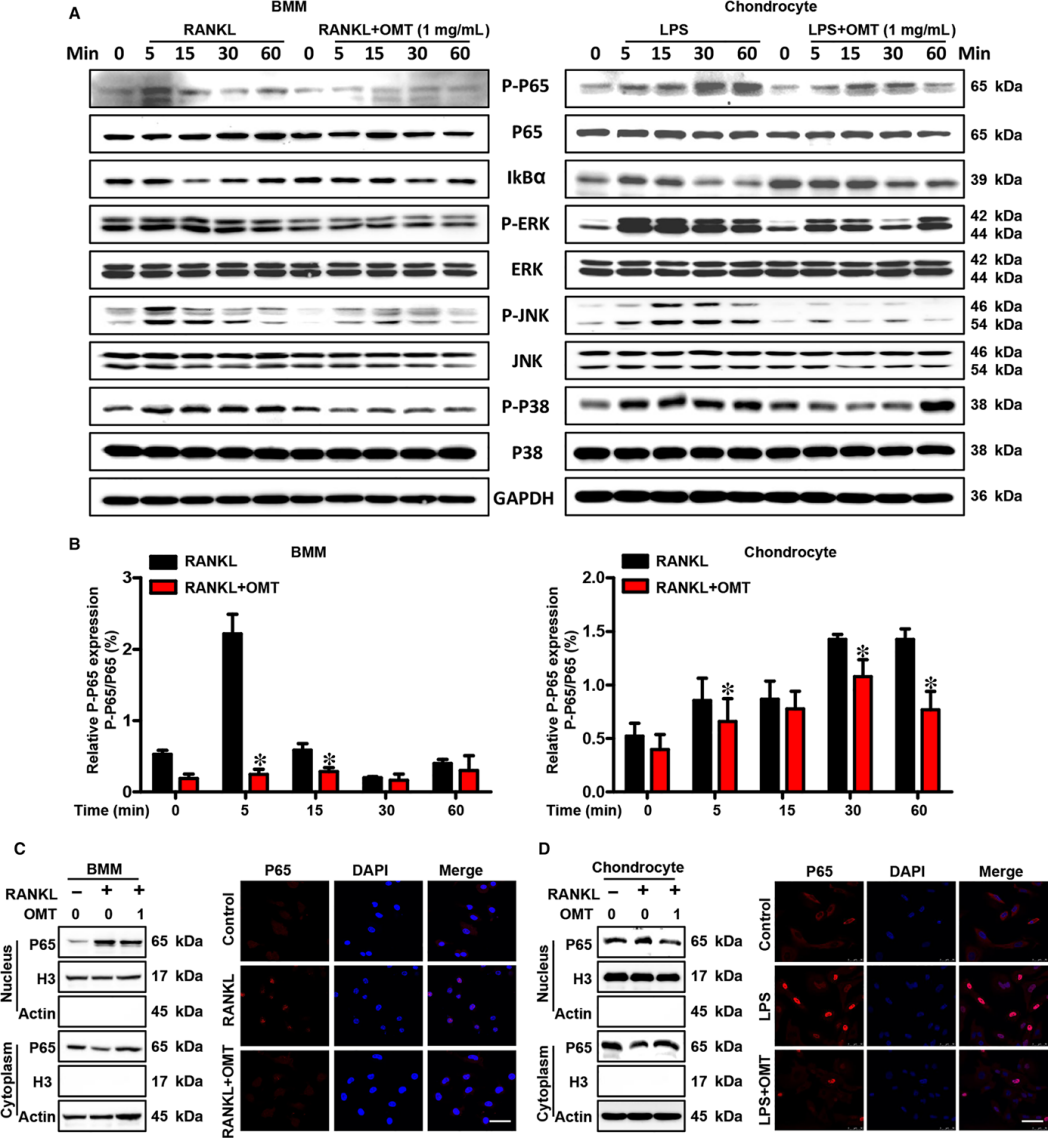
OMT attenuates NF‐κB and MAPK signalling pathway activation. A, The activity of NF‐κB and MAPK signalling pathways in mouse BMMs (or chondrocyte) was determined using Western blotting; GAPDH was used as the loading control. B, The ratio of p‐p65 to total p65 was quantified using ImageJ. The expression and nuclear translocation of NF‐κB p65 in chondrocytes (C) and BMMs (D) were determined using immunofluorescence and a nuclear‐cytoplasmic extraction assay. Scale bar, 10 μm. P65 protein expression levels in the nucleus and cytoplasm were analysed by Western blot. All experiments were performed 3 times. Values are expressed as the mean ± SD; *P < .05 compared to control

The NF‐κB signalling pathway is an important signal transducer involved in both LPS‐induced inflammation and RANKL‐induced osteoclastogenesis and has been implicated as a key regulator of cartilage destruction and bone remodelling in OA.^30^, ^31^ To determine the mechanism of OMT on chondrocyte inflammation, catabolism and osteoclastogenesis, BMMs (and human chondrocytes) were treated with RANKL (and LPS) in the presence or absence of 1 mg/mL OMT for 5, 15, 30 minutes or up to 1 hour, and the expression levels of p65, p‐p65 and IκBα were assessed using Western blotting. As shown in Figure 4A, the ratio of p‐p65 to p65 was higher in the absence of OMT at several time‐points (Figure 4B). At the same time, the immunofluorescence microscopy and nuclear‐cytoplasmic extraction results further supported that OMT suppressed the nuclear translocation of p65 (Figure 4C,D). The results indicated that OMT inhibited the activation of NF‐κB signalling pathway and may offer value as a novel alternative for treating NF‐κB related diseases.

MAPK is another signalling pathway involved in OA‐related cartilage destruction and OC‐mediated bone resorption.^32^ In our study, both RANKL and LPS significantly promoted the phosphorylation of ERK, JNK and p38 in human chondrocytes and BMMs, while OMT abolished these effects at several time‐point after stimulation (Figure 4A). Together, these data suggest that the inhibitory effect of OMT on chondrocyte inflammation and osteoclastogenesis may be due to the attenuation of the NF‐κB and MAPK signalling cascades.

### 3.5 OMT treatment rescues cartilage degeneration in a mouse ACLT OA model in vivo

**Figure 5:**
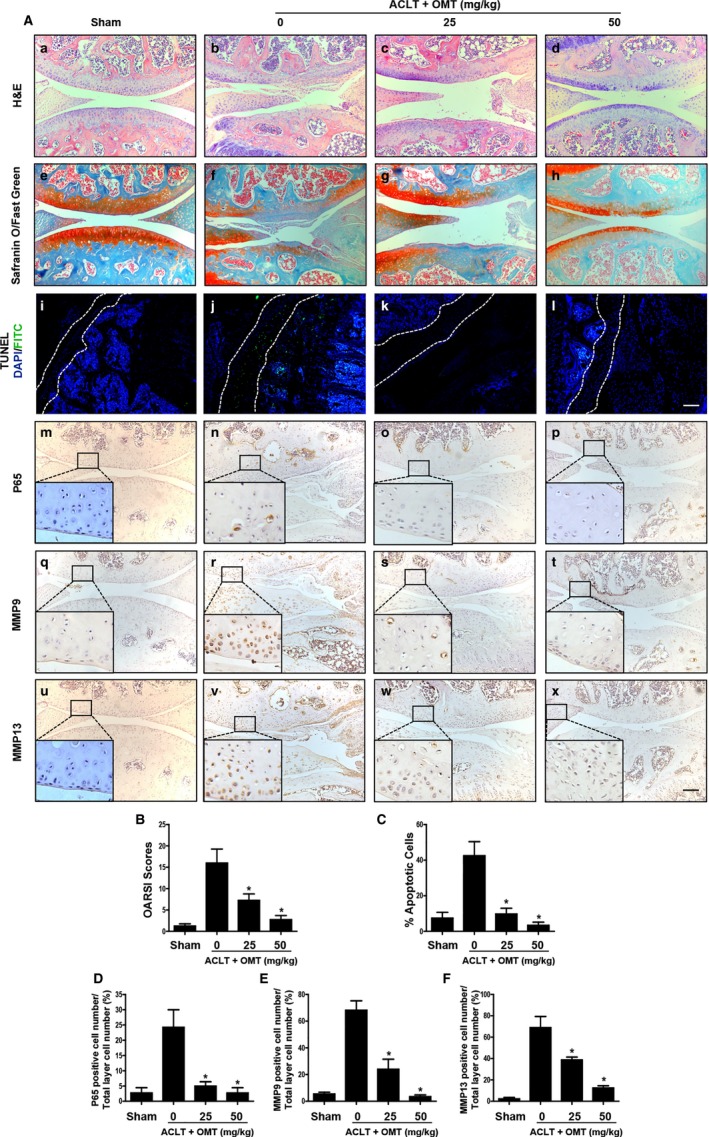
OMT protects against ACLT‐induced cartilage destruction in vivo. A, a‐d: H&E staining of cartilage and tibial subchondral bone. e‐h: Safranin O/Fast green staining in the articular cartilage. i‐l: Apoptotic cells in that articular cartilage were detected by TUNEL staining, the cell nuclei were stained with DAPI. m‐p: Immunohistochemical analysis of P65. q‐t: Immunohistochemical analysis of MMP9. u‐x: Immunohistochemical analysis of MMP13. B, The overall OARSI histological score was assessed. C, The percentage of apoptotic chondrocytes in cartilage was calculated. The panels show the quantification of D, p65; E, MMP9; F, MMP13 immunohistochemical staining. All scale bar, 100 μm. All values are given as the means ± SD *P <* *.05 compared with the ACLT‐operated group

To explore the potential protective effects of OMT on articular cartilage in vivo, we first performed a histological analysis using H&E and Safranin O/Fast green staining. The results showed that in PBS‐treated mice, there was a severe total erosion of cartilage, which was observed down to the subchondral bone in some cases, compared with that of the sham control, while OMT‐treated animals showed less severe destruction, as evidenced by a reduced loss of Safranin O staining and surface regularity (Figure 5A a‐h). The OARSI scoring method was used to measure structural cartilage changes in the medial tibial plateau of all samples (Figure 5B). All samples were assessed independently by 2 experienced observers blinded to the treatment groups. The 2 observers reached a consensus on each section.

We then examined the biological effects of OMT on chondrocyte apoptosis; the extent of cell death was assessed by TUNEL staining. As shown in Figure 5A i‐l, compared to the control mice, ACLT‐operated mice exhibited a significant increase in the number of TUNEL‐positive cells in the articular cartilage zone, while in the OMT‐treated group, there was less apoptosis in the cartilage chondrocytes. The percentage of apoptosis was calculated (Figure 5C).

The expression levels of MMP9 and MMP13 and the activation of NF‐κB signalling pathway in cartilage were detected using immunohistochemical staining. As shown in Figure 5A m‐p, the percentage of P65‐positive chondrocytes in the ACLT with PBS group was substantially higher than that in the sham surgery group, while no change was observed in the low‐dose and high‐dose OMT‐treated groups. Similarly, the expression levels of MMP9 and MMP13 were coordinated with that of p65 (Figure 5A q‐x). Based on the immunostaining intensities of 5 randomly selected areas of the articular cartilage, the expression levels of p65 (Figure 5D), MMP9 (Figure 5E) and MMP13 (Figure 5F) were calculated and analysed.

### 3.6 OMT treatment attenuates tibial subchondral bone loss in a mouse ACLT OA model in vivo

**Figure 6:**
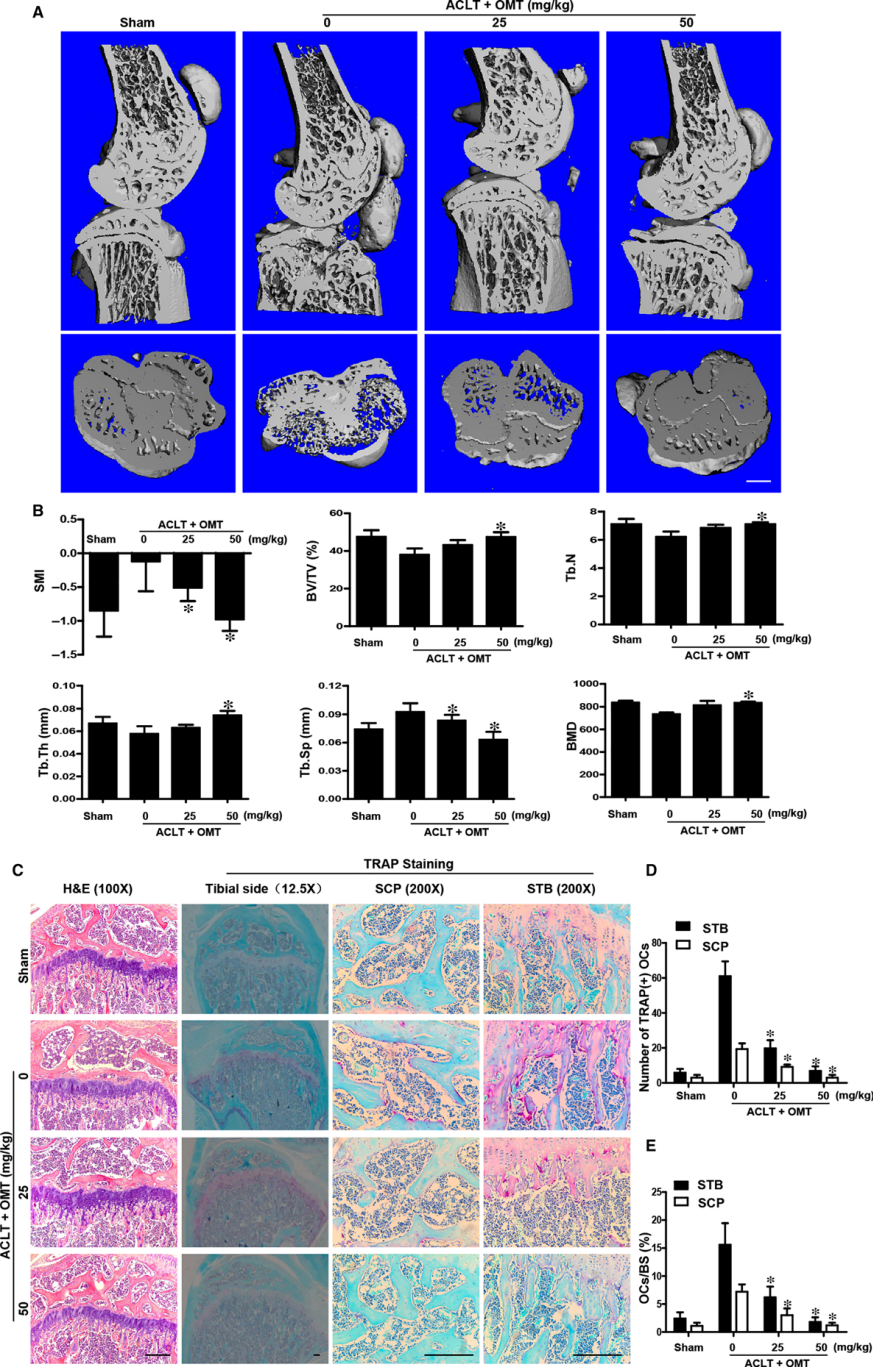
OMT protects against ACLT‐induced bone loss in vivo. A, Representative microCT 3D reconstructed images were obtained for each group. Scale bar, 1 mm. B, The BV/TV, BMD, Tb.Sp, Tb.Th, Tb.N and SMI were measured. C, The OCs in the subchondral plate (SCP) and subchondral trabecular bone (STB) were detected using H&E and TRAP staining (200×). Scale bar, 100 μm. The number of osteoclasts (D) and the percentage area (E) stained by TRAP in sections were analysed. All bar graphs are expressed as the means ± SD. *P <* *.05

Bone resorption was increased and resulted in bone loss in the post‐traumatic osteoarthritis (PTOA) animals.^33^ To assess the therapeutic potential of OMT for preventing tibial subchondral bone loss in vivo, the tibial subchondral bone of the mice was analysed by μCT. The 3‐dimensional (3D) reconstruction results revealed extensive subchondral bone resorption in the ACLT group, which was observed as extensive bone loss in the subchondral bone, when compared with that of the sham control group, while intraperitoneal injections of OMT in ACLT mice dramatically attenuated the tibial subchondral bone loss (Figure 6A). SMI, BV/TV, BMD, Tb.Th, Tb.N and Tb.Sp were measured according to the 3D‐reconstructed images (Figure 6B).

We next examined the effects of OMT on osteoclastogenesis in vivo using TRAP staining. As shown in Figure 6C, ACLT mice displayed an increased number of TRAP‐positive multinucleated cells in the subchondral plate (SCP) and subchondral trabecular bone (STB), while OMT treatment abolished those effects in the ACLT mice. The number of TRAP‐positive osteoclasts (Figure 6D) and the osteoclast surface/bone surface area (OcS/BS) (Figure 6E) were calculated. These observations suggested that OMT functioned as a strong inhibitor of osteoclastogenesis in vivo.

Finally, we examined the systemic toxicity of OMT, the body weights of mice were measured once a week over the treatment period. As shown in Figure S1, OMT showed no obvious damage on mice.

## 4 DISCUSSION

Osteoarthritis (OA) is a representative degenerative joint disease that affects not only articular cartilage but also subchondral bone. The crosstalk among osteochondral units is of great importance.^8^, ^34^, ^35^ Although pathological OA might selectively target a single joint tissue during the early stage of disease, both components of the joint will ultimately be affected because of their intimate association.

Articular cartilage is a complex tissue that is composed of chondrocytes as its unique cell type, which is embedded within an extracellular matrix. There is abundant evidence showing that chondrocytes alter their behaviour in OA. The reduced production of the cartilage matrix proteins aggrecan and collagen II and the increased expression of the cartilage degrading MMP enzyme and inflammatory cytokines are an important feature of OA. The degrading enzymes influence chondrocyte function to deviate away from normal homoeostasis and anabolism towards a catabolic phenotype, which leads to increased cartilage degradation.^3^, ^36^ In our study, we demonstrated that OMT inhibits LPS‐induced inflammation and catabolism in chondrocytes in vitro. Additionally, our ex vivo findings indicate that OMT protected cartilage against degradation partly by inhibiting MMPs and attenuates the LPS‐induced release of GAGs. These findings suggested that OMT may delay cartilage degeneration by suppressing the expression of degrading enzymes and maintaining the level of articular cartilage ECM.

Subchondral bone also plays a major role in maintaining articular cartilage integrity, and any attempt to repair a cartilage lesion without sufficient support from intact subchondral bone will likely result in failure.^37^ Because of the crosstalk between cartilage and subchondral bone, the integrity of articular cartilage has been proposed to depend on mechanical properties of the underlying bone.^38^ Nevertheless, in some cases, researchers view subchondral bone degradation as the causative factor in the development of OA, and studies based on animal models showed that during the early stages of OA, the subchondral bone has a decrease in both volume and stiffness.^39^ Osteoclasts, as the primary cells involved in bone resorption, were recently identified as an effective target for the prevention and treatment of osteolytic disorders. Based on these previous studies, we concluded that an effective treatment against OC‐mediated subchondral bone resorption should attenuate cartilage destruction. In our study, we demonstrated that the RANKL‐induced osteoclastogenesis was dose‐dependently attenuated by OMT administration. Additionally, OC marker gene expression, including that of TRAP, NFATc1, CTR and CTSK, was significantly inhibited by OMT treatment. These results indicated that OMT may be an attractive therapeutic agent for treating bone loss‐related diseases.

**Figure 7:**
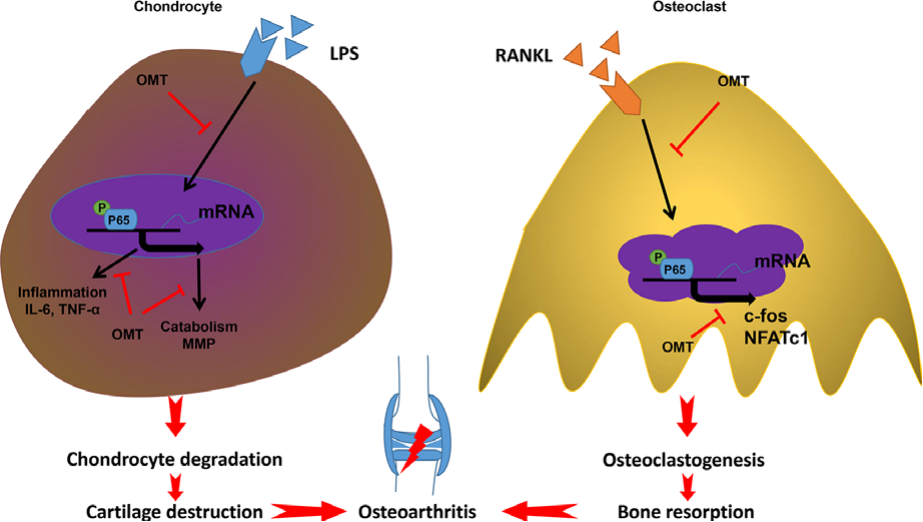
Proposed mechanism of OMT interference with the OA‐associated vicious cycle. OMT exerts dual effects on cartilage degradation and osteoclast‐mediated bone resorption, and those effects are imparted by inhibiting the activation of NF‐κB signalling pathways

MAPK and NF‐κB signalling cascades are 2 important signalling pathways in inflammatory reactions and osteoclastogenesis, which are required for the formation of osteoclasts and the production of inflammatory cytokines. Our results suggest that the anti‐osteoclastogenic and anti‐inflammatory effect of OMT may in part be the result of the inhibition of the NF‐kB and MAPK signalling cascades, and this inhibition leads to the down‐regulation of the master transcription factor for OC formation, chondrocyte inflammation and catabolism (Figure 7).

ACLT is one of the most common sporting injuries and is associated with an increased risk for developing post‐traumatic osteoarthritis (PTOA).^40^ The ACLT injury model has been shown to reliably reproduce the changes found in human osteoarthritic bone. In our study, we employed this model to explore the effects of OMT on OA cartilage and subchondral bone in vivo. The results demonstrated an increased remodelling of the knee joint subchondral bone during early stage OA that led to substantial bone loss and increased porosity. With disease progression, compared to the sham controls, ACLT mice displayed a significant loss of cartilage proteoglycans and exhibited a significantly higher degree of destruction, which was observed down to subchondral bone.

In summary, our results have shown, to our knowledge for the first time, that OMT, a plant‐derived natural molecule, dampens the catabolic response in cartilage and suppresses the production of pro‐osteoclastogenic mediators, thus resulting in a reduction in osteoclastogenesis. These dual effects of OMT in ACLT mice substantially attenuate the progression of OA. As a result, OMT interferes with the vicious degeneration cycle associated with OA and may be developed as a potential treatment alternative for OA in the future.

## CONFLICT OF INTERESTS

All authors declare that they have no conflict of interests concerning this article.
