# Supplementary figures and images for: Oxymatrine exerts protective effects on osteoarthritis via modulating chondrocyte homoeostasis and suppressing osteoclastogenesis
Source: J Cell Mol Med. 2018 May 25;22(8):3941–54. doi: 10.1111/jcmm.13674 (PMC6050479; doi:10.1111/jcmm.13674)

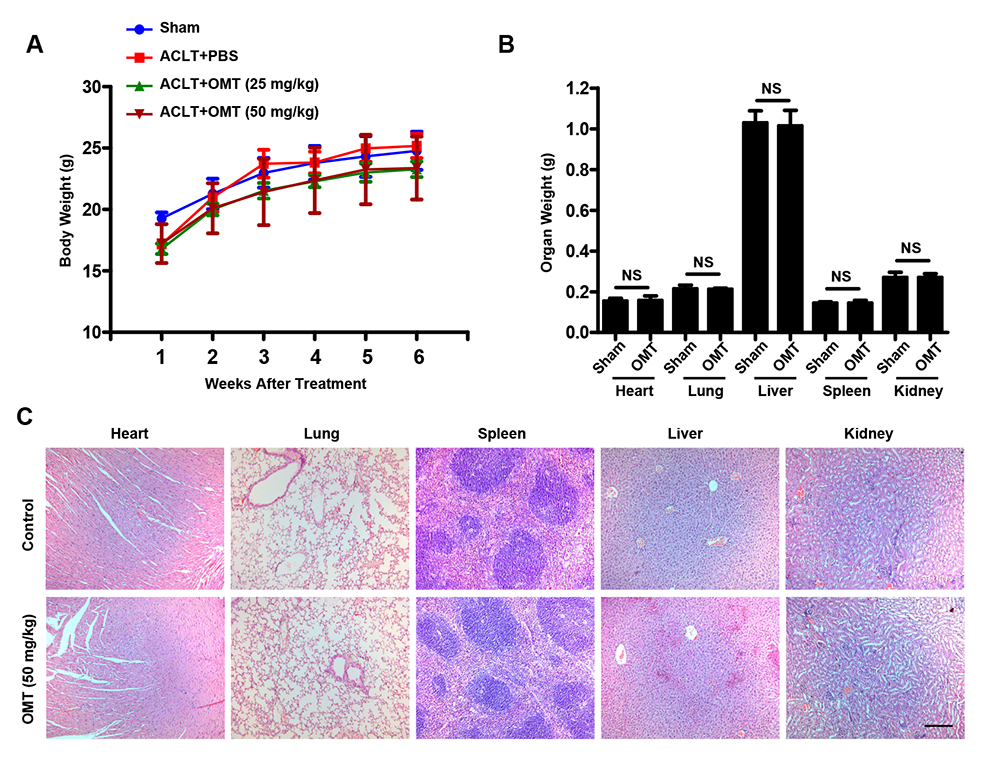

Supplement: Supplementary file 1 [file JCMM-22-3941-s001.tif]
