# Supplementary material for: Oxymatrine exerts protective effects on osteoarthritis via modulating chondrocyte homoeostasis and suppressing osteoclastogenesis
Source: J Cell Mol Med. 2018 May 25;22(8):3941–54. doi: 10.1111/jcmm.13674 (PMC6050479; doi:10.1111/jcmm.13674)
Supplement: Supplementary file 2 [file JCMM-22-3941-s002.docx]

Supplementary table.1

Primer sequences for real-time PCR studies

| Gene | Primers for real-time PCR |
| --- | --- |
| IL-6 | F: AACCTTCCAAAGATGGCTGAA  R: CCTCAAACTCCAAAAGACCAGTG |
| IL-8 | F: CTGGACCCCAAGGAAAACTG  R: CCCTACAACAGACCCACACAAT |
| TNF-ɑ | F: TCTTCTCGAACCCCGAGTGA  R: CCTCTGATGGCACCACCAG |
| MMP-2 | F: AACTACAACTTCTTCCCTCGCAA  R: CAAAGGCATCATCCACTGTCTCT |
| MMP-9 | F: AGTCCACCCTTGTGCTCTTCCC  R: TCTGCCACCCGAGTGTAACCAT |
| MMP-13 | F: CTTCACGATGGCATTGCTGAC  R: CGCCATGCTCCTTAATTCCA |
| CTSK | F: GGGAGAAAAACCTGAAG  R: ATTCTGGGGACTCAGAGAGC |
| TRAP | F: TGTGGCCATCTTTATGCT  R: GTCATTTCTTTGGGGCTT |
| CTR | F: TGCAGACAACTCTTGGTTGG  R: TCGGTTTCTTCTCCTCTGGA |
| DC-STAMP | F: CTTGCAACCTAAGGGCAAAG  R: TCAACAGCTCTGTCGTGACC |
| NFATc1 | F: TGGAGAAGCAGAGC  R: GCGGAAAGGTGGTATCTCAA |
| β-actin (human) | F: ACAGTCAGCCGCATCTTCTT  R: ACGACCAAATCCGTTGACTC |
| β-actin (mouse) | F: AGCCATGTACGTAGCCATCC  R: CTCTCAGCAGTGGTGGTGAA |
